# Transition to Virtual Care Services during COVID-19 at Canadian Pain Clinics: Survey and Future Recommendations

**DOI:** 10.1155/2023/6603625

**Published:** 2023-04-03

**Authors:** Victoria Borg Debono, Samuel Neumark, Norman Buckley, Ramesh Zacharias, Eleni Hapidou, Jennifer Anthonypillai, Susy Faria, Carrie-Lynn Meyer, Thomas Carter, Nadia Parker, Brenda Lau, Emmanuel Abreu, Scott Duggan, Etienne Bisson, Josie Pierre, Regina Visca, Patricia Poulin

**Affiliations:** ^1^Department of Health Research Methods, Evidence and Impact, McMaster University, 1280 Main Street West, Hamilton, Ontario L8S 4K1, Canada; ^2^Translational Research Program, Department of Laboratory Medicine and Pathobiology, Temerty Faculty of Medicine, University of Toronto, 263 McCaul Street, Toronto, Ontario M5T 1W7, Canada; ^3^Department of Anesthesia, McMaster University, 1280 Main Street West, Hamilton, Ontario L8S 4K1, Canada; ^4^Michael G. DeGroote Pain Clinic, Hamilton Health Science, 1200 Main Street W, Hamilton, Ontario L8N 3Z5, Canada; ^5^CBI Health Clinics, 272 Richmond Street E Suite 100, Toronto, Ontario M5A 1P4, Canada; ^6^CHANGEpain Clinic, 5655 Cambie Street Lower Level, Vancouver, British Columbia V5Z 3A4, Canada; ^7^Kingston Health Sciences Centre-Hotel Dieu Hospital Site, 166 Brock Street, Kingston, Ontario K7L 5G2, Canada; ^8^Operational Stress Injury (OSI) Clinic and Pain Management, Ste-Anne's Hospital, 305 Boul des Anciens-Combattants Sainte-Anne-de-Bellevue, Montreal, Quebec H9X 1Y9, Canada; ^9^McGill University Health Centre, Alan Edwards Pain Management Unit Montreal General Hospital, 1650 Cedar Ave, Montreal, Quebec H3G 1A4, Canada; ^10^Department of Anesthesia and Pain Medicine, University of Ottawa, 75 Laurier Ave. E, Ottawa, Ontario K1N 6N5, Canada

## Abstract

**Introduction:**

Due to the COVID-19 pandemic, healthcare centers quickly adapted services into virtual formats. Pain clinics in Canada play a vital role in helping people living with pain, and these clinics remained essential services for patients throughout the pandemic. This study aimed to (1) describe and compare the transition from in-person to virtual pain care services at Canadian pain clinics during the onset of the COVID-19 pandemic and (2) provide postpandemic recommendations for pain care services to optimize the quality of patient care.

**Materials and Methods:**

We used a qualitative participatory action study design that included a cross-sectional survey for data collection and descriptive analysis to summarize the findings. Survey responses were collected between January and March of 2021. The survey was administered to the leadership teams of 11 adult pain clinics affiliated with the Chronic Pain Centre of Excellence for Canadian Veterans. Responses were analyzed qualitatively to describe the transition to the virtual pain services at pain clinics.

**Results:**

We achieved a 100% response rate from participating clinics. The results focus on describing the transition to the virtual care, current treatment and services, the quality of care, program sustainability, barriers to maintaining virtual services, and future considerations.

**Conclusions:**

Participating clinics were capable of transitioning pain care services to the virtual formats and have in-person care when needed with proper safety precautions. The pandemic demonstrated that it is feasible and sustainable for pain clinics to have a hybrid of virtual and in-person care to treat those living with pain. It is recommended that moving forward, there should be a hybrid of both virtual and in-person care for pain clinics. Ministries of Health should continue to develop policies and funding mechanisms that support innovations aimed at holistic healthcare, interdisciplinary teams, and the expansion of clinics' geographical reach for patient access.

## 1. Introduction

At the onset of COVID-19 in Canada, there were significant reductions in access to care as in-person visits were dramatically restricted by provincial mandates [1]. In hospitals, staff was re-deployed into other more urgent areas [2] and medical centers quickly transitioned as many services as possible from in-person to virtual delivery to minimize the spread of infection [3]. This situation was a challenge for all pain clinics across Canada.

Chronic pain is a common disease affecting the lives of one in five Canadians [4]. The condition accounts for about 10–16% of emergency department visits and is expensive for our healthcare system [4]. Chronic pain is the most common cause of disability and has a total societal economic cost in Canada between $38.3 and 40.4 billion CAD in 2019 according to the Canadian Pain Task Force report from October, 2020 [4]. Despite the high prevalence of chronic pain in our communities, there are significant gaps in healthcare professional education [4], and optimal care is not generally accessible to many patients [5]. Pain clinics in Canada play a vital role in filling this gap for people living with pain and these clinics remained essential services for patients throughout the pandemic.

There is a need to understand pain clinics' transition to the virtual care to inform recommendations for future operations. The objectives of this study are to (1) describe and compare the transition from in-person to virtual pain care services at Canadian pain clinics during the onset of the COVID-19 pandemic and (2) provide postpandemic recommendations for pain care services to optimize the quality of patient care.

## 2. Materials and Methods

### 2.1. Study Design

We used a qualitative participatory action study design that included a cross-sectional survey for data collection and descriptive analysis to summarize the findings [6]. This research method was selected to maximize stakeholder involvement in the research process to ensure the relevancy of findings to the real-world contexts and provide actionable recommendations to positively impact pain care.

We surveyed seven leadership teams representing eleven pain clinics. One leadership team managed five clinics; a complete list of participating Canadian pain clinics can be found in Supplementary Material 1. We used a convenience sampling strategy [7] to recruit clinics affiliated with the Chronic Pain Centre of Excellence (CPCoE) for Canadian Veterans [8] that have previously expressed interest to participate in research initiatives that provide care to military veterans and the general adult population. This project was reviewed and approved by the Hamilton Integrated Research Ethics Board in Hamilton, Ontario, Project Number 11519.

### 2.2. Survey Development and Validity

The survey was developed by the principal investigator (PI), VBD, based on a facilitated focus group discussion with study participants and pilot testing. The purpose of the initial facilitated focus group was to ask what type of questions would be best to describe their clinics' transition to the virtual pain care service and how those services compare to in-person care. All discussion points were used by the PI to develop an initial version of the survey, which then went through a validity testing phase.

To assess face validity and content validity, we used methods described by Dillman [9, 10]. We circulated the survey to all the study participants to review and provide feedback during formal meeting discussions regarding its face validity. Content validity was assessed by two pain clinic medical directors to assess the alignment of survey questions with the overall study objective. After consultations, the PI integrated feedback from the validity assessments into the survey.

### 2.3. Data Collection

In early January, 2021, participants were contacted via email by the PI to explain the purpose of the study and the intended use of survey data. Using an adopted method suggested by Dillman to improve the survey response rate [9], we specifically followed up with participants frequently. Three reminder emails were sent to participants every two weeks until all responses were collected by March, 2021. Informed consent was provided and obtained on the first page of the electronic survey.

In the context of this research study, the term “service” refers to a variety of synchronous forms of care, such as standard appointments, interventions, individual/group programs, workshops, injection procedures, or rehabilitation services. Participants answered survey questions about the following domains: (a) observations of the transition to virtual pain services, (b) treatments/services provided, (c) sustainability and future capabilities, (d) barriers to maintaining virtual services, (e) facilitators for maintaining virtual service, (f) quality of care, (g) service impact, and (h) clinic knowledge of patient experience with care.

The survey included long-form open-ended questions to allow respondents to be as descriptive as they needed to fully answer each question. A full copy of the survey is provided in Supplementary Material 2 along with a description of the conditional branching logic. All information was collected through an online-based electronic survey, called Cognito forms [11], that inputted encrypted and password protected data into a Microsoft Excel ® database.

### 2.4. Data Analysis

By following an adapted qualitative descriptive analysis explained by Sandelowski [12], survey responses were analyzed, interpreted, and categorized into themes and subthemes. The survey data were extracted into a template worksheet to facilitate a holistic review. Repetitive topics and consistent responses were rearranged and organized into themes in a second summary worksheet. When numerical characteristics were reported by participants, they were counted and used as examples to support qualitative responses. After summarizing the data, the results were independently shared with the participants to verify the accuracy and relevancy prior to knowledge dissemination, an important step of participatory action research [13].

## 3. Results

By March, 2021, we achieved a 100% response rate from all participating clinics. All eleven clinics reported having pain care services transition from in-person to online formats. However, each clinic had different past experiences with offering virtual care services. While all clinics had to transition services to online formats, eight had some existing online services prior to COVID-19 and one chose to create brand-new services during pandemic. The characteristics of the participating clinics and their virtual services are detailed in Table 1. The following survey results focus on describing the transition to virtual pain care delivery, current treatment and services, the quality of care, program sustainability, barriers to maintaining virtual services, and future considerations.

### 3.1. Transition Observations

#### 3.1.1. Time to Implementation

All clinics identified the need to transition programs to a virtual format around mid-March 2020. There was variation in the time it took for services to make the transition. Some clinics (*n* = 3) expressed they were already equipped and experienced in providing services in a virtual format prior to the COVID-19 pandemic and were able to transition in a few days. For most clinics (*n* = 7), the transition took about 4 to 12 weeks. There were no appreciable differences between clinics that were hospital-based or community-based in the time required to set up virtual care services.

#### 3.1.2. Required Resources

The primary resource required by clinics for the transition to the virtual pain care was time for staff to adapt current resources to fit a virtual delivery format. Resource allocation for all clinics (*n* = 11) was a collaborative effort within their multidisciplinary teams. Clinical staff was consulted to create a virtual care delivery material, technical training, and safety training for staff and patients in accordance with COVID-19 protocols. Administrative and clinical staff was consulted to design the workflows for virtual referral intake processes, staff work schedules, billing requirements, appointment scheduling for patients, and quality improvement measures.

Redesigning workflow required assessing new technological platforms to identify appropriate resources to support patient care while incorporating privacy, confidentiality, stability, and threat analyses. Some of these platforms were Zoom ® for healthcare, Ontario Telemedicine Network ®, Reacts ®, and Microsoft Teams ®.

#### 3.1.3. Referring Physician Education

For most clinics (*n* = 8), education for referring physicians about virtual services was very similar to that provided for in-person services. About half of the clinics (*n* = 5) mentioned that referring physicians were provided with information on changes to clinics' management processes to enable remote service workflows, adopt new technologies, and how to remotely access patient support groups. Additionally, referring physicians were updated with information regarding on-site safety protocols implemented to reduce infection risk for procedures, physical exams, and active rehabilitation services.

#### 3.1.4. Funding Sources

Most of the in-person programs that made the transition to virtual formats are publicly funded, fully or in part, by their respective provincial governments through their Ministries of Health (MOH) through direct funding to clinics and/or funding for physician or other healthcare professional visits. Some aspects of care within those programs are not fully covered by provincial governments and require additional funding sources to support operations, such as government administered workers' compensation programs, correctional services programs, or Veteran Affairs Canada. Additionally, one clinic expressed that for psychological and social work, funding of salaried providers comes from charitable contributions, which help ensure there are no fees for this service to patients.

### 3.2. Treatment and Services Provided

#### 3.2.1. Overview of Services

The content of the services provided by the clinics varied, but collectively fell under two main categories: coordinated therapeutic programs and educational sessions, and workshops. There were a total of 72 services that transitioned to virtual delivery in the participating clinics (*n* = 11). Despite some variation between clinics regarding the types of services provided, in-person and virtual program offerings within clinics were described as very similar. Experiential and therapeutic components that could only be provided in-person were not offered virtually (e.g., auscultating patients during musculoskeletal assessments, some medical interventions, aquatic therapy, and forms of physical therapy). Due to the physical treatment limitations of virtual delivery, clinics expressed the importance of having in-person appointments for these components.

#### 3.2.2. Process of Virtual Care

The process for arranging virtual care services at participating clinics varied. Here is an example onboarding process described by one clinic. Patient care is coordinated by designated staff members from the start to the end of care. The staff assists patients with scheduling, technology requirements, bookings, and consultations, so patients are not alone throughout this process and are kept on track. Patients are booked at the beginning for an interprofessional assessment via the telephone to determine a care plan and explain the risks of online care. Then, a video call is made with the patient to ensure they are in a safe environment for group exercises when appropriate. A staff member helps with technological difficulties during sessions. Patients enrolled in the program are sent emails regarding class schedules, group expectations, and questionnaires. If a patient is deemed not appropriate or ready for a program, recommendations for other services are discussed with them at the end of the assessment. Each patient is generally paired with a case manager to provide ongoing support and treatment throughout the program.

#### 3.2.3. Integration into Patient's Existing Care Plan

Most clinics (*n* = 9) had established methods of integrating patients' pain care plans with other specialist clinics or with general practitioners. There were slight differences in this process between clinics; however, for the most part, the processes remained unchanged for clinics during the transition to virtual care. Two clinics reported using electronic medical records (EMR) to make treatment documentation efficient, tailored to the patient, easy to use, and accessible to multiple care providers. The use of EMRs helped clinicians coordinate care and facilitate sharing information in the online environment.

#### 3.2.4. Tracking and Evaluating Services

Most clinics (*n* = 10) use the standardized questionnaire data to monitor treatment outcomes, track adherence, and evaluate program effectiveness. Clinics that reported using paper questionnaires needed to convert them to secure online formats during the transition to virtual care. The online questionnaires still enabled clinics to collect patient demographic characteristics and several pain-related measures. The ability to administer questionnaires with virtual care delivery enabled clinics to continue using data for program evaluation, quality assurance, care planning, identifying treatment options, and measuring treatment satisfaction. To specifically track program effectiveness, most clinics were able to collect data about the duration of treatments, number of virtual visits versus in-person, treatment completion rates, cancelation or no-show rate, and patient functional outcomes. Overall, the feasibility of monitoring program effectiveness for quality improvement was extended from in-person to virtual care by capturing relatively the same metrics.

### 3.3. Quality of Care

#### 3.3.1. Patient Care

Most clinics (*n* = 9) were able to share that overall patient care and response to care were the same before and after the transition to virtual care. Three clinics found that treatment completion rates, functional outcomes, and client satisfaction were the same. The length of appointments was shorter for virtual-only services compared to that of in-person or a hybrid of the two; however, the clinics suggested that virtual-only be used for engaged patients and when there is no need to treat or examine the patient in-person. It was also noted by one clinic that online participation increased compared to in-person. According to the leadership teams, patients were overall pleased with accessing pain care virtually as it eliminated travel; however, some are still experiencing barriers due to not having a home environment conducive to participate online or not having the technology.

#### 3.3.2. Screen Time Limits

There was variation between clinics with screen time limits during treatment because of differences in provided programs or treatments. One clinic did not have a limit imposed but reported hearing many conversations about screen fatigue. The inability to stand or change position during in-person education sessions was a suggested explanation. It is consistent among clinics that they provide hourly breaks to minimize screen fatigue.

#### 3.3.3. Group Psychology Sessions

Some clinics (*n* = 5) have expressed that group psychology in-person and virtual sessions are more similar than different, with one clinic suggesting their services were equivalent in both. However, technology access issues for some patients pose a barrier to receive care. Some patients have reported to their clinicians that they find it more difficult to develop group cohesion in a virtual setting. With that said, this was noted to be largely dependent on the dynamics in each group of patients. Additionally, it was remarked by another clinic that in-person has the advantage to respond better to nonverbal cues and can promote more participation. One clinic reported having smaller groups for virtual care compared to in-person (e.g., 5 versus 20 in-person); however, virtual attendance is increasing as clinics develop more ways to promote engagement and socialization. Some other in-person group benefits that are lost using a virtual platform include collegiality between participants, socialization, and group cohesion.

### 3.4. Program Sustainability

All clinics reported there were no substantial changes in their staff and clinician capacity to support their existing patients in all services that transitioned to virtual care. Although, one clinic mentioned that there were no extra resources to provide individual pain management and therapy for those not wanting to participate in group programming, which is the bulk of coordinated therapeutic programs provided. It is also important to note there was considerable variability in the clinics' capacity to support their services in general at baseline.

For example, after the transition to virtual care, individual clinics were able to support between 6 and 15 patients in their group coordinated therapeutic programs at a time and up to 100 people in their education workshops. One-on-one patient care capacity for the individual clinics varied from 1600 to 20,000 patients treated per year. All clinics reported that they had the capacity to train new staff and clinicians, if recruitment was successful, irrespective of programs transitioning from in-person to the virtual care.

### 3.5. Barriers to Maintain Virtual Services

#### 3.5.1. System Barriers

There were several concerns raised by participating clinics regarding infrastructural barriers for creating or maintaining virtual services. For example, one clinic reported that approximately 15% of their patients do not have access to the Internet or appropriate technology (e.g., laptop, speakers, and camera) for virtual care. Another barrier mentioned was the inability to perform the physical examination required for proper patient assessment, evaluation, and treatment. Over time, the expected increase in in-person appointments may decrease the ability of clinics to continue, offering virtual care because of competition for clinical resources. Clinics also reported that a lack of funding to support the maintenance and expansion of programs for virtual or in-person delivery will be a constant barrier.

#### 3.5.2. Patient Experience Barriers

In one clinic, the primary concern some patients have is the length of time spent in front of a screen, even with the breaks provided, and especially for those who suffer from headaches. The nature of virtual care also led to varying degrees of physical and mental tolerance for a sitting position in education sessions. Clinics reported that some patients prefer a human connection with an on-site group. In particular, it was an observation and opinion of a few veteran facing clinics that veterans feel more comfortable starting face-to-face and then transitioning to virtual care. There was also great variance in reported trust from patients of virtual platforms with respect to privacy and confidentiality. Lastly, it was noted that many patients do not have appropriate home environments to participate in services, such as a private, safe, and quiet space to engage in care.

#### 3.5.3. Scalability of Virtual Services

Clinics varied greatly in their sense of their ability to scale up virtual services and increase future capacity. While one clinic had confidence in their ability to grow in capacity for service delivery, others shared one or more of the following concerns: (a) provider and staff availability to support patient demand and attention needed for each patient for one-on-one care, (b) continued increases in the need for technological support for virtual care, (c) the need for better self-scheduling of programs for patients, (d) the need to eliminate MOH billing rules for frequency of patient sessions, and (e) more MOH funding needed overall particularly for the nonmedical services required for complex pain care.

#### 3.5.4. Privacy Concerns

No significant privacy concerns were identified by the clinics regarding their operations. All indicated they were compliant with all provincial standards, the Personal Health Information Protection Act, and the Freedom of Information and Protection of Privacy Act. In addition, clinics indicated that they were compliant with regulations set by their associated hospitals and health professional regulatory colleges. Some clinics (*n* = 3) mentioned issues were on the patients' end. It was shared that some patients had concerns related to not having private spaces to do a virtual appointment or general online privacy concerns.

### 3.6. Considerations for Maintaining Virtual Care

#### 3.6.1. Key Personnel

Five clinics expressed that they were able to manage the transition to virtual care with their existing personnel. However, many clinics (*n* = 7) mentioned that they struggled to hire a new psychologists, social workers, physiotherapists, and occupational therapists for their rehabilitation programs. The lack of funding and lack of available allied health professionals negatively impacted the clinics' abilities to grow and expand care to address the overwhelming demand for their pain services.

#### 3.6.2. Demand for Services

Demand for services increased for many clinics (*n* = 6) or stayed constant for the others after the start of the COVID-19 pandemic and the transition to the virtual care. One clinic group, with multiprovince community-based locations, reported that veteran demand for services appeared to be the highest in Ontario, then Quebec, Atlantic provinces, Alberta, British Columbia, and then Saskatchewan in descending order. Another community-based clinic indicated that from May to September, 2019, 3500 appointments were completed; whereas, in the same time frame in 2020, over 26,000 single patient appointments were completed. Additionally, another clinic shared that wait-times have increased for care overall and continue to increase because of the COVID-19 pandemic. One clinic (*n* = 1) expressed that currently, the 90^th^ percentile wait-time from receipt of referral to first visit is 9 months and growing, which is way above their pre-COVID time of 3 months. The demand for the use of a virtual platform was not formally measured; however, anecdotally, one clinic reported there are about equal proportions of patients wanting virtual and in-person services. It was also noted that the geographic reach of services greatly expanded posttransition to virtual care and that most programs offered could now provide care province-wide.

#### 3.6.3. Future Considerations for Virtual Service Development

Only one clinic (*n* = 1) indicated that funding was in place for the future development of programs, virtual or in-person. The other clinics had funding concerns that impacted future development. For example, some clinics expressed that MOH funding structure needs change so clinics can hire more staff and patients can attend more visits.

Some clinics (*n* = 5) shared various factors that could help ensure there is funding for future development after the transition to virtual services: (a) Flexibility after the pandemic to support virtual platforms to provide care to patients when appropriate; (b) allow patients to receive access to services in different provinces if that province has a clinic or services with the needed expertise; (c) convert site specific-siloed funding requests for program creation into expanded collaborations with provincial partners to share the program design and services where possible; (d) MOH support for a provincial virtual care delivery platform and integration with EMRs; (e) maintain ongoing funding to support the positions that are currently available; and (f) cost recovery from third party payers to allow for increased capacity for services that are not a part of the base funding from MOH eligible services.

One clinic shared some alternative perspectives to better support the future development of effective virtual pain care services, where we must consider looking beyond funding to see if we can implement the following: (i) Create a network of shared standardized resources and programs developed with quality assurance criteria; (ii) open services that can be shared between sites and regions which will maximize staffing and potentially reduce wait times with some programming; (iii) develop a high quality and empowering system where patients can begin self-managed treatment while they wait to see a pain specialist physician; and (iv) develop services that target chronic pain prevention with an acute pain stream targeting injuries that have a higher likelihood of leading to chronic pain.

## 4. Discussion

The objectives of this study were to describe and compare the transition from in-person to virtual care services at pain clinics across Canada and provide postpandemic recommendations for pain care services to optimize the quality of patient care. This survey found that participating pain clinics were remarkably quick in their processes to implement adaptations, allowing most medical services and programs to be provided in a virtual format when appropriate. The response to the pandemic and our results demonstrate that a hybrid model of virtual and in-person care is feasible for those living with pain and is further supported in a study by Cascella et al. [14].

### 4.1. Feasibility of Virtual Care

We found that clinics already had what they needed to transition many of their programs into a virtual format for pain care delivery. Regardless of how clinics were funded, most clinics were able to work with their team to transition most programs and forms of care into an online format within a span of 4 to 12 weeks. This rapid transition to virtual care is comparable to many other jurisdictions in North America [15, 16]. For example, a pediatric chronic pain clinic in Ontario transitioned all their appointments over two weeks [17]. Similar to other pain clinics [17], our participants required improving infrastructure such as a stable and secure online platform (e.g., Zoom for Healthcare) with multiple user capability, funds to purchase the licenses for online platform use, and high-speed Internet service with large data packages.

Our study also found that patients reported difficulties developing group cohesion in virtual settings. This challenge of maintaining group cohesion is supported by the study by Lopez et al. [18] that compared group cohesion between in-person dialectical behavior therapy and video teleconferencing. Even with the limitations of group cohesion for video conferencing, the authors note that virtual group therapy is a feasible alternative to in-person group therapy [18].

A discussion about virtual care in any context requires consideration for patients without adequate privacy and compatible technology to receive proper care. The pandemic reminded the healthcare community about the digital divide [19, 20] and how those with lower socioeconomic status may not be able to engage in safe and effective virtual care [21]. It is important for pain clinics across Canada to address equity and accessibility concerns while maintaining and expanding their virtual care operations.

Regarding the general processes for appointments, they remained mostly unchanged when integrating pain care into a patient's existing care plan, integrating patient questionnaire data, and tracking program effectiveness when applicable. Overall, the continuation of providing virtual pain care services is feasible with the current resources, while understanding certain procedures will always require in-person visits.

### 4.2. Sustainability of Virtual Care

In addition to being feasible, the delivery of virtual care was also sustainable for now and in the future. For the most part, clinics expressed that their current funding from MOH and potential grants would sustain telemedicine efforts, but additional grants from the MOH are needed for future developments and innovations to address the increasing demand for services. All clinics described having enough staff to support the virtual delivery of care and train new staff and clinicians. This situation is not surprising considering that the pandemic started at a time in history when our technology (e.g., the Internet, computers, and videoconferencing platforms) had the capacity and capability to adapt to increases in the volume of virtual meetings. This situation was similar across jurisdictions with many health systems making seamless transitions to virtual care [15, 16].

The virtual delivery of pain care services can and will likely continue in the future, with the notion that there should be in-person care when needed or requested by the patient or when there are technological limitations to service delivery. Some patients do not have a home environment conducive to participating in virtual care (e.g., safe and quiet private spaces in their home). Others do not trust or feel comfortable with online platforms with respect to privacy and confidentiality and some do not have the technology available to them to participate in online care and others. These privacy concerns are reported throughout published literature [22] and have been described extensively in telemedicine guidelines for years [23].

Furthermore, there are notable cost savings [24] and improved efficiencies [25] for both patients and clinics with virtual care, supporting its sustainability. On the patients' end, virtual care allows them to save transportation costs, time, and ease in getting care, especially for those with mobility issues [26]. For clinics, virtual care allows clinicians to have a greater geographical reach.

### 4.3. Recommendations Post-Pandemic

Based on the findings of this survey, we summarized key postpandemic recommendations for pain clinic virtual care services in Table 2. These recommendations may also apply to other types of clinics that provide care for both specialized and chronic conditions.

### 4.4. Strengths and Limitations

One of the strengths of this study is the detailed survey questions. The open-ended questions allowed pain clinics to provide detailed information regarding their experience transitioning to the virtual care and suggest recommendations for future developments. Additionally, we had a diverse group of participants which strengthened the perspectives in our data. The pain clinics represented many geographic regions of Canada and operational settings. Clinics were located in British Columbia, Alberta, Ontario, Quebec, and the Maritimes, as well as, hospital-based, university-affiliated, and community-based settings. Our sampling frame included clinics part of the CPCoE network and represents clinics that are leaders and experts in pain care in Canada. They provided comprehensive and valuable information to share about the phenomena of interest in this study.

One of the limitations of this study was the length of the survey. Though it provided detailed and valuable information, a substantial time commitment was needed to answer many of the open-ended questions which may have reduced the quality of responses. Considering the collected data were also self-reported, the participants may have been prone to recall bias [27]. Another limitation is the sample size of our participating clinics. It would have been ideal to recruit more clinics to participate in the study, but due to significant COVID-19 restrictions, overall staffing shortages, and complex logistics, we were limited in scope.

### 4.5. Future Directions

The recommendations made for pain clinics postpandemic are likely to apply to other pain clinics in the country and other clinics that provide care to patients with chronic conditions. They are also consistent with recommendations from the Canadian Pain Task Force [28]. However, to uncover any outstanding and unique needs and experiences, the next steps would include doing a member check with other pain clinics and clinics treating patients with chronic conditions in Canada to see if the results of this study are also applicable to them and their experience during the pandemic.

More research is also required to examine the cost savings and efficiencies associated with a hybrid model for both the patient and the clinics. Additionally, MOHs need to consider the allowance for all the patients to receive and access services in different provinces, especially if another province has a clinic or services with the necessary expertise for that patient and has components that can be accessed online. This would also allow increased access to pain services expertise, which is particularly relevant for veterans because of their federal healthcare funding. In this way, pain service support can be spread geographically to truly improve access and early intervention and reduce disparity in pain care access across the country.

## 5. Conclusion

The COVID-19 pandemic presented many challenges for medical clinics across the country. This survey found that participating clinics were capable and remarkably quick in their processes of transitioning pain care services to virtual formats and having in-person care when needed with proper safety precautions. The pandemic demonstrated that it is feasible and sustainable for pain clinics to have a hybrid of virtual and in-person care to treat those living with pain.

It is recommended that moving forward beyond the pandemic, there should be a hybrid of both virtual and in-person care for pain clinics. Ministries of Health should continue to develop the policies and funding mechanisms that support innovations aimed at holistic healthcare, interdisciplinary teams, and the expansion of clinics' geographical reach for patient access.

## Figures and Tables

**Table 1:** Characteristics of participating pain clinics (*n* = 11).

Clinic settings	Number of clinics (%)
Hospital-based	5 (45)
Community-based	6 (55)

Province	Number of clinics (%)
British Columbia	2 (18)
Alberta	2 (18)
Ontario	3 (27)
Quebec	2 (18)
New Brunswick	1 (9)
Nova Scotia	1 (9)

Types of virtual services^*∗*^	Number of services
Existing (prior to pandemic)	9
Transitioned (during pandemic)	72
New (after onset of pandemic)	4

^*∗*^“services” refers to a variety of synchronous forms of care, such as standard appointments, interventions, individual/group programs, workshops, or rehabilitation services.

**Table 2:** Postpandemic recommendations for virtual care services at pain clinics.

Key recommendations
(1) Clinics should provide a hybrid model of virtual and in-person care: Virtual care is feasible, sustainable, and of equal quality for most components of care and pain management. Some patients perform well having their initial consult in-person and then move to virtual care when desirable and feasible for both the patient and the healthcare provider
(2) Funding agencies need to increase support and provide grants to pain clinics: Provinces' respective Ministries of Health or alternate funding agencies need to provide grants that will allow pain clinics to innovate and perform quality improvement assessments to better serve their patients virtually and in-person
(3) Patients should have the right to choose: Patients should be allowed to exercise personal preference to request and receive in-person care even if the treatment or care can be delivered virtually. Some patients will require in-person care and not every patient has a home situation conducive to receiving virtual care
(4) In-person care must remain for services involving experiential and therapeutic components that require one's physical presence: Some components of care cannot be delivered virtually and require in-person care delivery. For example, physical examinations, auscultating patients during musculoskeletal assessments, medical interventions, aquatic therapy, or physical therapy
(5) Allow patients to access services in different provinces: If another province has a clinic or services with the needed expertise, patients should be allowed to receive virtual care remotely. In this way, pain care services can be spread geographically and utilized beyond provincial borders and the flagship pain centers. This change would improve pain care access and early intervention
(6) Training and investing in more interdisciplinary healthcare teams: Training more licensed psychologists, social workers, physiotherapists, and occupational therapists will help support pain clinic operations, expand rehabilitation services, and provide more generalized pain care to meet the demands of the population. Increased student recruitment for professional mental health programs in conjunction with more pain specialty training within training programs may help improve the availability and enhance virtual care delivery methods

## Data Availability

The survey responses (data) used to support the findings of this study have not been made available because of the need to maintain participant confidentiality as supported by the Hamilton Integrated Research Ethics Board in Hamilton, Ontario. Responses may have contained identifying participant information; thus, raw survey response data will not be available.

## Acronyms

CPCoE: Chronic Pain Centre of Excellence for Canadian Veterans
MOH: Ministry of Health
PI: Principal investigator
EMR: Electronic medical record.

## Supplementary Materials

Supplementary Materials Supplementary Material 1: List of participating pain clinics. Supplementary Material 2: Copy of study survey for data collection.
