# Supplementary material for: Transition to Virtual Care Services during COVID-19 at Canadian Pain Clinics: Survey and Future Recommendations
Source: Pain Res Manag. 2023 Apr 3;2023:6603625. doi: 10.1155/2023/6603625 (PMC10085656; doi:10.1155/2023/6603625)
Supplement: Supplementary Materials — Supplementary Material 1: List of participating pain clinics. Supplementary Material 2: Copy of study survey for data collection. [file 6603625.f1.docx]

**Supplementary Material 1:** List of Participating Canadian Pain Clinics

| **Name** | **Location** |
| --- | --- |
| Ottawa Hospital Pain Clinic & Ottawa Hospital Rehabilitation Centre Chronic Pain Management Program | Ottawa, Ontario |
| Kingston Health Sciences Centre - Hotel Dieu Hospital Site Chronic Pain Clinic | Kingston, Ontario |
| Hamilton Health Sciences Michael G. DeGroote Pain Clinic | Hamilton, Ontario. |
| Ste Anne de Bellevue (Veteran Facing) Pain Clinic | Montreal, Quebec. |
| McGill University Health Centre, Alan Edwards Pain Management Unit | Montreal, Quebec. |
| CHANGEpain Clinic | Vancouver, British Columbia. |
| CBI Health Pain Clinics | a. Calgary, Alberta;  b. Edmonton, Alberta;  c. Halifax, Nova Scotia;  d. Victoria, British Columbia;  e. Moncton, New Brunswick. |

**Supplementary Material 2: Survey**

**Page 1**

**A Survey of Online Pain Care Services at Canadian Pain Clinics**

Dear Participating Pain Clinics,

Your help is greatly appreciated in sharing details about your online pain care services which are being initiated as a result of current restrictions on face-to-face visits in medical clinics. Thank you for taking the time to fill out this survey!

This survey is being conducted by Dr. Victoria Borg Debono, Department of Anesthesia at McMaster University in affiliation with the Chronic Pain Centre of Excellence for Canadian Veterans (CPCoE).

This survey will contain a series of questions related to your online pain services to patients and may take some time to complete.

Only aggregated data or information will be published. Information collected from this survey will be used for academic publication purposes. Although the CPCoE is not engaged in the provision of care, its research mandate includes assessing ways in which care can be provided to veterans, some of whom are living in underserviced or distant areas and may prefer to access virtual care for some or all of their pain care. The intent is to also collect information that can be put into a publishable form and which can provide necessary and useful information for the CPCoE and Veterans Affairs Canada. You will not be asked for any identifying information such as ages, names of people or patient case details.

If you have any questions about this study, please contact Victoria Borg Debono (xxxxx@xxxx.xx). For information about your rights as a participant in this study, please contact the Hamilton Integrated Research Ethics Board at XXX XXX XXXX, Extension XXXXX.

The software we are using for this survey is provided by Cognito Forms. They operate under the conditions outlined by Cognito Forms Privacy Statement as stated here: https://www.cognitoforms.com/legal/privacy

Your participation in this survey is completely voluntary, and you may exit this survey at any time by exiting your browser window. Please note that your answers will only be saved once you click the final submit button. Dr. Victoria Borg Debono would have sent you the list of questions in an advance email so you will be able to fill them out here fully and submit. If you choose to withdraw from this study after consenting, please contact Dr. Victoria Borg Debono at (xxxxx@xxxx.xx) and all your information provided in the survey will be securely deleted and will not be used for this study.

By selecting Next below, you are consenting to participate in this survey.

Next

**Page 2**

Which clinic are you answering for?  Answer required

How many services, programs or forms of care do you have that were in-person that have now been converted to an online format post-COVID-19 face-to-face or in-person visit restrictions? Answer required

How many services, programs, or forms of care do you have that were or are in-person but were already offered in an online format before COVID-19 face-to-face in-person visit restrictions? Answer required

How many services, programs, or forms of care that you currently offer online are brand new, in that they were originally designed from scratch to be online and were not based on anything you provided in-person?  Answer required

Next

***Aside note of survey*** ***conditional logic branching.***

**Conditional logic note: Participants will see Page 3 when they meet this conditional logic:**

**Answer to question** *How many services, programs, or forms of care do you have that were in-person that have now been converted to an online format post-COVID-19 face-to-face or in-person visit restrictions?* **is greater than 0**

**Conditional logic note: Participants will see Page 4 when they meet this conditional logic:**

**Answer to question** *How many services, programs, or forms of care do you have that were or are in-person but were already offered in an online format before COVID-19 face-to-face in-person visit restrictions?*  **is greater than 0**

**Conditional logic note: Participants will see Page 5 when they meet this conditional logic:**

**Answer to question** *How many services, programs, or forms of care that you currently offer online are brand new, in that they were originally designed from scratch to be online and were not based on anything you provided in-person?* **is greater than 0**

**Page 3**

On page 2 of this survey, you indicated that you have services, programs, or forms of care that were or are in-person that have now been converted to an online format post-COVID-19 face-to-face or in-person visit restriction. Please answer the following questions related to those types of services, programs, and forms of care to the best of your ability. If a question does not apply to your clinic(s), please say so.

**Please name each of these services, programs, or forms of care.***Answer required*

*For example, if you stated you had 3 of these types of services, programs, and forms of care, please name each on a separate line.*

*1) Program 1 Name*

*2) Program 2 Name*

*3) Program 3 Name*

**For each service, program, or form of care mentioned above please indicate how each one is funded.**

*E.g. For each program, indicate the following:*

*1) For Program X*

*a) Who provides the funding for the service, such as a third party or provincial government, patient insurance.*

*b) What is the model of funding? e.g. Fee-for-service, a fee per patient, overall program funding, are there any salaried providers and who supports those salaries, or some combination of these funding sources.*

**For each service, program, or form of care mentioned please indicate the time it took from the identified need to have the program or form of care in an online format to when patients could receive the care online.**

*e.g. For Program X: We first knew that Program X needed to be offered online around March 10th, 2020. From that point in time, it took about 3 weeks to get program X fully offered online. By March 31st, 2020, Program X was fully offered online.*

**For each service, program, or form of care mentioned above that you now offer online, what were the resources required to get that program delivered and offered into an online format?** (i.e. please describe the technological (web conference platform used, etc.), programming, personnel, and financial resources that were necessary to create the content, organize the content, and deliver the program to patients online.)

**Please describe, if applicable, the type of education provided to the referring physician for each service, program, or form of care that you now offer online.**

**For each service, program or form of care named above please describe the content of the program (in person and online, separately) by speaking to the items such as Medication Management, Education, Counselling, Physical Therapy and anything else provided. Are the in person and online versions similar or different in any way? Please describe.**

**For each service, program or form of care mentioned above please describe how it is integrated into patient's existing care plan either with their GP, other treatments they are undertaking with you or at another clinic.**

**For each service, program, or form of care mentioned above, if applicable, please describe the integration and use of questionnaire data from the minimum data set.**

*For instance, are you using questionnaires to ask how the patient is doing at different time points and to monitor patient compliance? If so, please describe what these questionnaires ask, what they are for, and how you use the information.*

**For each service, program or form of care mentioned above, if applicable, please describe how you are tracking or monitoring online program effectiveness.**

*i.e. This can include monitoring effectiveness regarding future prevention of pain, peri-operative optimization, Veteran specific care, rehabilitation, mental health, and chronic pain.*

**Is there funding for future service development and if so, where is that money coming from? Please describe.**

*e.g. MSP/OHIP/Other provincial health, program funding, extended health, interprovincial reciprocal agreements*

**For each service, program, or form of care mentioned above that are now online do you have enough staff and clinicians to support currently enrolled patients in each of your online programs? What is your current capacity for each online service, program, or form of care mentioned above (e.g. number of patients you can support)?**

**For each service, program, or form of care mentioned above, do you have enough staff and clinicians to support increased demand for the program?**

**For each service, program, or form of care mentioned above, please describe the capacity you have to train new staff and new clinicians.**

**Please describe any barriers you foresee having that may prevent you from continuing to provide each service, program, or form of care mentioned above.**

**What is the scalability of the service provided and mentioned above?**

*e.g. Are you able to grow each of the services? Are they as big as it can be? etc.*

**If applicable, what are the conceptual or philosophical objections for each service, program, or form of care mentioned above?**

*e.g. culture*

**If applicable, what issues are there with funding do you see that can prevent the future development of each service, program, or form of care mentioned above?**

I**f applicable, are there any privacy restrictions that act as a barrier to services, programs, or forms of care mentioned above?**

*e.g. institutional, governmental, etc.*

**What is the access like to obtain needed personnel for each service, program or form of care mentioned above? Please describe.**

**If applicable, what are the conceptual or philosophical supports for each service, program or form of care mentioned above? Please describe.**

*e.g. culture*

**Are there any facilitating factors that will ensure that there is funding for the future development each service, program or form of care mentioned above?**

**How is each service, program or form of care mentioned above promoted and who promotes it? Please describe.**

**What is the patient demand like for each service, program or form of care mentioned above?**

**If available, in your professional view, please describe overall what the patient care and response to care were like before (in-person) and after the introduction of virtual care services for each service, program, or form of care mentioned above?**

*e.g. regarding patient satisfaction; standardized measures of progress (e.g. minimal dataset as from national registry) or as per your existing program Quality Assurance process if present.*

**What is the screen time limits for patients for each service, program, or form of care mentioned above?**

**If applicable, please compare what group psychology is like in person vs. online.**

**In your view, how is the quality of care affected or different for each service, program or form of care mentioned above when a GP unattached vs attached?**

**Please compare, if applicable or possible, the quality of care of services, programs or forms of care mentioned above that have modules of treatment that are coordinated and managed by staff for the patient vs. ones where the patient has to manage and set-up their appointments.**

**Please compare, if applicable or possible, the quality of care of services, programs or forms of care mentioned above that have A) a combination of individual care and group care vs. B) just individual care vs. C) just group care.**

**Please compare, if applicable or possible, the quality of care of services, programs or forms of care mentioned above that have access to A) extended health covered services vs. B) just program funding vs. C) just provincial health care (e.g. OHIP), vs. D) some combination of these funding sources.**

**If possible, please describe the number of patients who have been referred to each service, program, or form of care mentioned above since its start.**

**If possible, please describe the number of patients who have been inquiring about each service, program or form of care mentioned above since its start.**

**If possible, please describe the number of patients who have been accepted into each service, program, or form of care mentioned above since its start.**

**If possible, please describe the number of patients who have been treated in each service, program, or form of care mentioned above since its start.**

**If possible, please describe the geographical reach of each service, program or form of care mentioned above.**

**What is the number of patients without a GP referral for each service, program or form of care mentioned above?**

**If applicable, please describe the number of sessions taken by patients for each service, program or form of care mentioned above. Also, if applicable and possible please describe the number of sessions retaken by patients for each service, program or form of care mentioned above.**

**If possible and applicable, please describe the types of patients you have for each service, program or form of care mentioned above. Describe the best you can.**

*For example, age range, gender, sex, employed, unemployed, retired, veterans, Automobile accidents, Workers Compensation Board, Long Term Disability etc.*

**Please describe the type of education you provide to the patient in each service, program or form of care that you now offer online.**

**In your view, please describe what the patient expectations are from each service, program or form of care that you now offer online.**

**In your view, please describe the patient experience is for each service, program, or form of care provided online regarding the following: Registration, Patient Setup, Ongoing Patient Support, Information provided to them about the process, and Patient engagement during the appointment.**

**In your professional experience and if applicable, have there been any patient objections with each service, program or form of care mentioned above? If so, what are they?**

**In your professional experience and if applicable, have there been any issues with patients lack of access to necessary technology for each of the services, programs, or forms of care mentioned above that have come to your attention? (e.g. Internet service, devices, equipment) If so, please describe it.**

Next

**Page 4**

On Page 2 of this survey you indicated that you have services, programs or forms of care that were or are in-person but were already offered in an online format prior to COVID-19 face-to-face in-person visit restrictions. Please answer the following questions related to those type of services, programs and forms of care to the best of your ability. If a question is not applicable to your clinic(s), please say so.

**Please name each of these services, programs or forms of cares.** *Answer required*

*For example, if you stated you had 3 of these types of services, programs and forms of care, please name each on a separate line.*

*1) Program 1 Name*

*2) Program 2 Name*

*3) Program 3 Name*

**For each service, program or form of care, please indicate how each one is funded.**

*E.g. For each program, indicate the following:*

*1) Name of Program X*

*a) Who provides the funding for the service, such as a third party or provincial government, patient insurance.*

*b) What is the model of funding? e.g. fee for service, fee per patient, overall program funding, are there any salaried providers and who supports those salaries, or some combination of these funding sources.*

**For each service, program or form of care mentioned above that you offer online, what were the resources required to get that program delivered and offered into an online format? (i.e. please describe the technological (web conference platform used, etc.), programming, personnel, and financial resources that were necessary to create the content, organize the content and deliver the program to patients online.)**


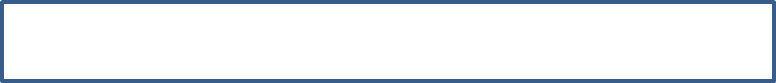


**Please describe, if applicable, the type of education provided to the referring physician for each service, program or form of care that you now offer online.**

**For each service, program or form of care named above please describe the content of the program (in person and online, separately), by speaking to the items such as Medication Management, Education, Counselling, Physical Therapy and anything else provided. Are there any differences and similarities between the in-person version of the program(s) and the online version(s)? Please describe.**

**For each service, program or form of care mentioned above please describe how it is integrated into the patient's existing care plan either with their GP, other treatments they are undertaking with you or at another clinic.**

**For each service, program or form of care mentioned above, if applicable, please name the questionnaires used and describe the integration or use of questionnaire data from a minimum data set.**

*For instance, are you using questionnaires to ask how the patient is doing at different time points and to monitor patient compliance? If so, please describe the names of these questionnaires, what these questionnaires ask, what they are for and how you use the information.*

**For each service, program or form of care mentioned above, if applicable, please describe how you are tracking or monitoring online program effectiveness.**

*i.e. This can include monitoring effectiveness regarding future prevention of pain, peri-operative optimization, Veteran specific care, rehabilitation, mental health, and chronic pain.*

**Is there funding for future service development and if so, where is that money coming from? Please describe.**

*e.g. MSP/OHIP/Other provincial health, program funding, extended health, interprovincial reciprocal agreements*

**For each service, program or form of care mentioned above that are now online do you have enough staff and clinicians to support currently enrolled patients each of your online programs?**

**What is your capacity at the moment for each online service, program or form of care mentioned above (e.g. number of patients you are able to support)?**

**For each service, program or form of care mentioned above, do you have enough staff and clinicians to support increased demand for the program?**

**For each service, program or form of care mentioned above, please describe the capacity you have to train new staff and new clinicians.**

**Please describe any barriers you foresee having that may prevent you from continuing to provide each service, program or form of care mentioned above.**

**What is the scalability of each of the services mentioned above?**

*e.g. Are you able to grow the services? Are they as big as they can be? etc.*

**If applicable, what are the conceptual or philosophical objections for each service, program or form of care mentioned above?**

*e.g. culture*

**If applicable, what issues are there with funding do you see that can prevent the future development each service, program or form of care mentioned above?**

**If applicable, are there any privacy restrictions that act as barrier to the services, programs or forms of care mentioned above?**

*e.g. institutional, governmental, etc.*

**What is the access like to obtain needed personnel for each service, program or form of care mentioned above? Please describe.**

**If applicable, what are the conceptual or philosophical supports for each service, program or form of care mentioned above? Please describe.**

*e.g. culture*

**Are there any facilitating factors that will ensure that there is funding for the future development each service, program or form of care mentioned above?**

**How is each service, program or form of care mentioned above promoted and who promotes it? Please describe.**

**What is the patient demand like for each service, program or form of care mentioned above?**

**If available, in your professional view, please describe overall what the patient care and response to care was like before (in-person) and after the introduction of virtual care services for each service, program or form of care mentioned above?**

*e.g. regarding patient satisfaction; standardized measures of progress (e.g. minimal dataset as from national registry) or as per your existing program Quality Assurance process if present.*

**What is the screen time limits for patients in each service, program or form of care mentioned above?**

**If applicable, please compare what group psychology is like in person vs. online for each of the programs mentioned above.**

**In your view, how is the quality of care affected or different for each service, program or form of care mentioned above when a GP unattached vs. attached?**

**Please compare, if applicable or possible, the quality of care of services, programs or forms of care mentioned above that have modules of treatment which are coordinated and managed by staff for the patient vs. ones where the patient has to manage and set-up their own appointments.**

**Please compare, if applicable or possible, the quality of care of services, programs or forms of care mentioned above that have a combination of A) individual care and group care vs. B) just individual care vs. C) just group care.**

**Please compare, if applicable or possible, the quality of care of services, programs or forms of care mentioned above that have access to A) extended health covered services vs. B) just program funding vs. C) just provincial health care (e.g. OHIP), vs. D) some combination of these funding sources.**

**If possible and in your estimation, please describe the number of patients who have been referred to each service, program or form of care mentioned above since its program start.**

**If possible and in your estimation, please describe the number of patients who have been inquiring about each service, program or form of care mentioned above since its program start.**

**If possible, please describe the number of patients who have been accepted into each service, program or form of care mentioned above since its program start.**

**If possible, please describe the number of patients who have been treated in each service, program or form of care mentioned above since its program start.**

**If possible, please describe the geographical reach of each service, program or form of care mentioned above.**

**What is the number of patients without a GP referral for each service, program or form of care mentioned above?**

**If applicable, please describe the number of sessions taken by patients for each service, program or form of care mentioned above. Also, if applicable and possible please describe the number of sessions retaken by patients for each service, program or form of care mentioned above.**

**If possible and applicable, please describe the types of patients you have for each service, program or form of care mentioned above. Describe the best you can.**

*For example, age range, gender, sex, employed, unemployed, retired, veterans, Automobile accidents, Workers Compensation Board, Long Term Disability etc. for each program or service or form of care mentioned above.*

**Please describe the type of education you provide the patient in each service, program or form of care that you mention in this section that are offered online.**

**In your view, please describe what the patient expectations are for each service, program or form of care mentioned above that you offer online.**

**In your view, please describe what the patient experience is for each service, program or form of care provided online regarding the following: 1) Registration, 2) Patient Setup, 3) Ongoing Patient Support, 4) Information provided to them about the process, and 5) Patient engagement during the appointment.**

**In your professional experience and if applicable, have there been any patient objections or concerns with each service, program or form of care mentioned above that have come to your clinic's attention? If so, what are they?**

**In your professional experience and if applicable, have there been any issues with patient lack of access to necessary technology for each of the services, programs or forms of care mentioned above that have come to your clinic's attention? (e.g. Internet service, devices, equipment) If so, please describe.**

Next

**Page 5**

On Page 2 of this survey you indicated that you have services, programs or forms of care that you currently offer online that are brand new, in that they were originally designed from scratch to be online and were not based on anything you provided in-person. Please answer the following questions related to those type of services, programs and forms of care to the best of your ability. If a question is not applicable to your clinic(s), please say so.

**Please name each of these services, programs or forms of cares.**

*E.g. For each program, indicate the following:*

*1) Name of Program X*

*a) Who provides the funding for the service, such as a third party or provincial government, patient insurance.*

*b) What is the model of funding? e.g. fee for service, fee per patient, overall program funding, are there any salaried providers and who supports those salaries, or some combination of these funding sources.*

**For each service, program or form of care mentioned above that you offer online, what were the resources required to get that program delivered and offered into an online format? i.e. please describe the technological (web conference platform used, etc), programming, personnel, and financial resources that were necessary to create the content, organize the content and deliver the program to patients online.**

**Please describe, if applicable, the type of education provided to the referring physician for each service, program or form of care that you offer online.**

**For each service, program or form of care named please describe the content of the program by speaking to the items such as Medication Management, Education, Counselling, Physical Therapy and anything else provided.**

**For each service, program or form of care mentioned above please describe how it is integrated into the patient's existing care plan either with their GP, other treatments they are undertaking with you or at another clinic.**

**For each service, program or form of care mentioned above, if applicable, please name the questionnaires used and describe the integration and use of questionnaire data from a minimum data set.**

*For instance, are you using questionnaires to ask how the patient is doing at different time points and to monitor patient compliance? If so, please name the questionnaires used, describe generally what these questionnaires ask, what they are for and how you use the information.*

**For each service, program or form of care mentioned above, if applicable, please describe how you are tracking or monitoring online program effectiveness.**

*i.e. This can include monitoring effectiveness regarding future prevention of pain, perioperative optimization, Veteran specific care, rehabilitation, mental health, and chronic pain.*

**Is there funding for future service development for these online programs and if so, where is that money coming from? Please describe.**

*e.g. MSP/OHIP/Other provincial health, program funding, extended health, interprovincial reciprocal agreements*

**For each service, program or form of care mentioned above that are now online do you have enough staff and clinicians to support currently enrolled patients in each of your online programs?**

**What is your current capacity for each online service, program or form of care mentioned above (e.g. number of patients you can support in each program)?**

**For each service, program or form of care mentioned above, do you have enough staff and clinicians to support an increased demand for the program?**

**For each service, program or form of care mentioned above, please describe the capacity you have to train new staff and new clinicians if the need should arise.**

**Please describe any barriers you foresee having that may prevent you from continuing to provide each service, program or form of care mentioned above.**

**What is the scalability of these online services mentioned above?**

*e.g. Are you able to grow the service? It is as big as it can be? etc.*

**If applicable, what are the conceptual or philosophical objections for each service, program or form of care mentioned above?**

*e.g. culture*

**If applicable, what issues are there with funding do you see that can prevent the future development each service, program or form of care mentioned above?**

**If applicable, are there any privacy restrictions that act as barriers to the services, programs or forms of care mentioned above? Please describe.**

*e.g. institutional, governmental, etc.*

**What is the access like to obtain needed personnel for each service, program or form of care mentioned above? Please describe.**

**If applicable, what are the conceptual or philosophical supports for each service, program or form of care mentioned above? Please describe.**

*e.g. culture*

**Are there any facilitating factors that will ensure that there is funding for the future development each service, program or form of care mentioned above?**

**How is each service, program or form of care mentioned above promoted and who promotes it? Please describe.**

**What is the patient demand like for each service, program or form of care mentioned above?**

**If available, please describe overall what patient care and response to care is like after introduction of virtual care service for each service, program or form of care mentioned above?**

*e.g. regarding patient satisfaction; standardized measures of progress (e.g. minimal dataset as from national registry) or as per your existing program Quality Assurance process if present.*

**What is the screen time limits for patients for each service, program or form of care mentioned above?**

**If applicable, please describe what group psychology is like online for patients or each service, program or form of care mentioned above.**

**In your view, how is the quality of care affected or different for each service, program or form of care mentioned above when a GP unattached vs attached?**

**Please compare, if applicable or possible, the quality of care of services, programs or forms of care mentioned above that have modules of treatment which are coordinated and managed by staff for the patient vs. ones where the patient has to manage and set-up their own appointments.**

**Please compare, if applicable or possible, the quality of care of services, programs or forms of care mentioned above that have A) a combination of individual care and group care vs. B) just individual care vs. C) just group care.**

**Please compare, if applicable or possible, the quality of care of services, programs or forms of care mentioned above that have access to A) extended health covered services vs. B) just program funding vs. C) just provincial health care (e.g. OHIP), vs. D) some combination of these funding sources.**

**If possible, please describe the number of patients who have been referred to each service, program or form of care mentioned above since it's start.**

**If possible, please describe the number of patients who have been inquiring about each service, program or form of care mentioned above since it's start.**

**If possible, please describe the number of patients who have been accepted into each service, program or form of care mentioned above since it's start.**

**If possible, please describe the number of patients who have been treated in each service, program or form of care mentioned above since it's start.**

**If possible, please describe the geographical reach of each service, program or form of care mentioned above.**

**What is the number of patients without a GP referral for each service, program or form of care mentioned above?**

**If applicable, please describe the number of sessions taken by patients for each service, program or form of care mentioned above. Also, if applicable and possible please describe the number of sessions retaken by patients for each service, program or form of care mentioned above.**

**If possible and applicable, please describe the types of patients you have for each service, program or form of care mentioned above. Describe the best you can.**

*For example, age range, gender, sex, employed, unemployed, retired, veterans, automobile accidents, Workers Compensation Board, Long Term Disability etc. for each service, program or form of care mentioned above.*

**Please describe the type of education you provide to the patient in each service, program or form of care mentioned above that you offer online.**

**In your view, please describe what the patient expectations are from each service, program or form of care that you now offer online mentioned above.**

**In your view, please describe what the patient experiences are for each service, program or form of care provided online regarding the following: 1) Registration, 2) Patient Setup, 3) Ongoing Patient Support, 4) Information provided to them about the process, and 5) Patient engagement during the appointment.**

**In your professional experience and if applicable, have there been any patient objections with each service, program or form of care mentioned above that have come to your clinic's attention? If so, what are they?**

**In your professional experience and if applicable, have there been any issues with patient lack of access to necessary technology for each of the services, programs or forms of care mentioned above that have come to your clinic's attention? (e.g. Internet service, devices, equipment) If so, please describe.**

Submit

Back

*Thank you for filling out the form. Your response has been recorded.*
